# Iodine Deficiency Disorders as a Predictor of Stunting among Primary School Children in the Aseer Region, Southwestern Saudi Arabia

**DOI:** 10.3390/ijerph18147644

**Published:** 2021-07-18

**Authors:** Fuad I. Abbag, Saeed A. Abu-Eshy, Ahmed A. Mahfouz, Mohammed A. Alsaleem, Safar A. Alsaleem, Ayyub A. Patel, Tarek M. Mirdad, Ayed A. Shati, Nabil J. Awadalla

**Affiliations:** 1 Department of Child Health, College of Medicine, King Khalid University, Abha 61421, Saudi Arabia; fabbg@kku.edu.sa (F.I.A.); ashati@kku.edu.sa (A.A.S.); 2 Department of Surgery, College of Medicine, King Khalid University, Abha 61421, Saudi Arabia; saeed@kku.edu.sa; 3 Department of Family and Community Medicine, College of Medicine, King Khalid University, Abha 61421, Saudi Arabia; mabade@kku.edu.sa (M.A.A.); asalslim@kku.edu.sa (S.A.A.); njgirgis@kku.edu.sa (N.J.A.); 4 Department of Clinical Biochemistry, College of Medicine, King Khalid University, Abha 61421, Saudi Arabia; ayyub@kku.edu.sa; 5 Department of Orthopedics, College of Medicine, King Khalid University, Abha 61421, Saudi Arabia; tmmirdad@kku.edu.sa

**Keywords:** iodine deficiency disorders, stunting, Saudi Arabia

## Abstract

Objectives: To investigate the present occurrence of stunting and explore the role of iodine deficiency disorders (IDDs) as a predictor of stunting among primary school children in the Aseer Region. Methods: In a cross-sectional investigation on school children in the Aseer region, thyroid enlargement was evaluated clinically. Urine was collected to evaluate iodine content. Results: The present study involved 3046 school-age pupils. The study disclosed a total goiter rate of 24.0% (95% CI: 22.5–25.5%). The median urinary iodine content (UIC) was 17.0 µg/L. A prevalence of stunting (height for age z score of less than −2) of 7.8% (95% CI: 6.9–8.8%) was found. In a logistic regression model, pupils having clinical goiter (aOR = 1.739; 95% CI: 1.222–2.475) and students having UIC of less than 17 µg/L (aOR = 1.934; 95% CI: 1.457–2.571) were considerably related with stunting. In the receiver operating characteristic (ROC) curve, urinary iodine content to forecast stunting was good (AUC = 0.611, 95% CI: 0.594–0.629). The curve recognized the optimum cutoff point of urinary iodine content to be ≤19.0 µg/L. The sensitivity was 59.66% (95% CI: 53.1–66.0) and the specificity was 57.62% (95% CI: 55.8–59.5). *Conclusion:* The present study showed that stunting among school-aged children presents a mild public health problem. On the other hand, a severe iodine deficiency situation was revealed among school children in the Aseer region. Continuous monitoring of iodine status among school children is therefore necessary. Concerted interventions that blend nutrition-sensitive with nutrition-specific approaches are expected to influence decreasing stunting significantly.

## 1. Introduction

Linear development malfunction in early years is the utmost predominant type of undernutrition universally. An anticipated 165 million persons under five years are stunted, with a height-for-age z-score (HAZ) lower than −2 (i.e., more than two standard deviations below the population median). However, a more significant amount of young people with HAZ -2 still have insufficient linear development and are consequently suffering from stunting [1,2]. Stunting more persistently delays the developmental capacity and human resources of people owing to its prolonged term influence on intellectual function and adult financial efficiency; it is consequently considered the best proxy indicator of health disparities among young people [3] and stunting has been of considerable global health importance [4]. The WHO aims to decrease stunting by 40% by 2025 [5]. While remarkable improvement has been achieved in Asia, with a decrease in the percentage of stunted children from 49% to 28% between 1990 and 2010, in Africa, the stunting occurrence has continued stationary around 40% and, due to population expansion, the amount of stunted children is rising [1].

The effects of child stunting are both instantaneous and long-term. They involve: raised morbidity and mortality; inadequate child growth and learning capability; intensified risk of infections and non-communicable diseases; increased liability to store fat mainly in the central region of the body; reduced fat oxidation; lower energy expenditure; insulin resistance; a greater risk of acquiring diabetes, hypertension, and dyslipidemia; lowered working capacity; and unfavorable maternal reproductive outcomes in adulthood. Furthermore, stunted children who underwent rapid weight gain after two years have an increased risk of becoming overweight or obese later in life [6,7]. In an extensive analysis of ten studies in Asia, Africa, and South America, there was a sound dose-response association between HAZ and death. However, potential confounders were not appropriately considered [8].

The trace element iodine is a fundamental constituent of thyroid hormones. Iodine occurs biologically in soil, water, plants, and animals. Humans take up iodine via routine dietetic consumption. Iodine concentrations in water and soil vary extensively across the world. Mountain areas, delta, and flood regions are biologically predisposed to low iodine concentrations. Inadequate dietary iodine consumption and associated deficiencies in thyroid hormones cause a group of unfavorable consequences called iodine deficiency disorders (IDDs) [9]. IDDs are chief public health complications across the world. A projected 2 billion persons globally are at risk of IDDs; 266 million are school-aged children [9]. On the other hand, there is a strong biological foundation for the responsibility of iodine in child development. Development failure is the chief expression of undernutrition during gestation and in childhood and is recognized by a height or weight that is too little compared with that of an average-fed reference population (stunting and underweight) or a weight that is too low for a given height (wasting) [10]. Iodine deficiency damages the production of thyroid hormones, including growth hormone expression [11]. The effect of growth hormones is enabled by insulin-like growth factor I, which is commonly conjugated to insulin-like growth factor binding protein-3 in circulation [12].

The Aseer Region, with an overall population of 1,200,000, lies in southwestern Saudi Arabia. A nationwide epidemiological survey of school children for IDDs [13] identified a modest status of IDDs in all areas of the Kingdom with reasonably higher prevalence for the Aseer Region.

The hypothesis tested is that if a child is suffering from iodine deficiency disorder, then they may be stunted, with a height for age z score of less than −2. The objectives of the present work were to explore the existing frequency of stunting and explore the role of IDDs as a predictor of stunting among primary school children in the Aseer Region.

## 2. Materials and Methods

### 2.1. Design

Cross-sectional investigation.

### 2.2. Target Population

School-age pupils aged 8–10 years residing in the Aseer region (for their high susceptibility to IDDs) [14].

### 2.3. Sampling and Field Activities

Applying the WHO guidelines handbook [15], at 95% confidence interval with a conventional approximation of the expected proportion of 45% [13], and with an absolute precision of 2%, the required size is computed to be 2377 pupils. To counterweigh for a potential loss, a total of 3000 pupils was considered to be contained in the investigation.

The procedures applied included sampling using stratified proportional allocation. First, the sample was selected, bearing in mind gender, the magnitude of the people in each region, rural–urban disparities, height above sea level, and governmental and private differences.

Personal communications were sent to pupils’ guardians, clarifying the investigation reason and requesting their written permission. Inclusion criteria: All children aged 8–10 years old in the region. Exclusion criteria: Those children who were absent during the survey or those whose fathers were not willing to include their children in the study.

### 2.4. Clinical Goiter

Inspection and palpation procedures were used. Criteria of the WHO for grading were used [16]. Grades were: Grade “0—no palpable or visible goiter; Grade “1”—a bulk in the neck in harmony with an enlarged thyroid that is palpable but not visible when the neck is in the neutral position; it also moves up in the neck upon swallowing; and Grade “2”—a mass in the neck noticeable when the neck is in a neutral position and is in harmony with an enlarged thyroid when the neck is palpated. The total goiter rate (TGR) is the summation of grades 1 and 2. To prevent inter-observer dissimilarity, thorough training and standardized techniques were implemented.

### 2.5. Measurement of Urinary Iodine Concentration

Casual urine samples were obtained from the pupils. They were provided with 40-mL plastic containers and asked to half-fill the containers with urine. These containers were tightly closed with plastic screw caps to prevent leakage and evaporation and were labelled with stickers for identification.

Urine samples were examined for iodine concentration using ammonium persulfate to process the samples, followed by the Sandel–Kolthoff reaction [17]. Calibrators, urine samples, and urine controls were added to 16 × 100 mm glass tubes, followed by the addition of 1.0 mL of 1 mol/L ammonium persulfate to all tubes. All samples were oxidized for 30 min in a 91–95 °C heating block. The samples were then cooled to room temperature and 2.0 mL of arsenious acid (0.0253 mol/L), 1.0 mL of 1.25 mol/L H2SO4, and 1.0 mL water were sequentially added. The tubes were then placed in a 32 °C water bath and incubated for 10 min. The reaction was started by adding 0.5 mL of ceric ammonium sulfate to all tubes, which were incubated precisely for 10 min. At the end of incubation, the percent transmission was read at 420 nm in a 10 mm light path cuvette. Water was used to adjust the spectrophotometer to 100% transmission.

The WHO standards [18] for evaluating the severity of iodine nutrition grades based on the median urinary iodine concentration (UIC) were applied. Quality control of iodine measurements was performed.

### 2.6. Assessment of Stunting

Weight and standing height were evaluated by proficient workers using standard procedures. They were documented to the closest 0.5 kg for weight and the nearest 1 cm for height. Stunting was described as height for age and sex that is two standard deviations (SDs) or more under the WHO standard median (–2 z-score or below) [19]. z score was computed using “WHO AnthroPlus for Personal Computers” [20].

### 2.7. Data Analysis

Data were investigated using the SPSS Software, version 22 (IBM Corp., released 2013, IBM SPSS Statistics for Windows, Version 22.0. Armonk, NY, USA) and “MedCalc” statistical software version 16.4.3 (MedCalc Software bv, Ostend, Belgium; https://www.medcalc.org, accessed on 21 March 2021). Frequency, percentage, arithmetic mean, median, standard deviation, and 95% confidence intervals were used to present the results. Suitable tests of significance were used at the 5% level. In addition, multivariable binary logistic regression analysis was computed to assess the adjusted odds ratio and the 95% confidence intervals to examine stunting associations.

A receiver operating characteristic (ROC) curve was generated through “MedCalc” software to predict the role of urinary iodine in identifying stunting. It showed the functioning of the cutoff points in terms of sensitivity versus 1-specificity. The area under the curve (AUC) is an assessment of the accuracy of the cutoff point. It lies between 0.5 and 1. Values < 0.6 indicate a weak classifier, and 1 shows an exceptional classifier.

## 3. Results

The current analysis involved 3046 pupils (1501 males and 1545 females).

### 3.1. Clinical Goiter Rate

Results disclosed that 18.5% (563) had grade I goiter, and 5.5% (167) had grade II goiter giving a total goiter rate (TGR) of 24.0% (95% CI: 22.5–25.5%).

### 3.2. Urinary Iodine Content

The average urinary iodine content (UIC) of the study sample of pupils was 19.03 ± 11.1 µg/L. The median UIC of the study sample was 17.0 µg/L.

### 3.3. Height-for-Age z Scores and Stunting

The height for age z score (HAZ) ranged from −4.75 to 3.5, with an average of −0.0588 ± 1.452 and a median of −0.09. The study showed that 238 school-aged children have HAZ of less than −2, giving a prevalence of stunting of 7.8% (95% CI: 6.9–8.8%).

### 3.4. Stunting Correlates

Table 1 shows that stunting was more frequent among females (8.3%, 129) than males (7.3%, 109). Yet it was not statistically significant (*p* = 0.17). On the other hand, stunting was significantly (*p* = 0.037) more frequent among children having clinical goiter (9.5%, 69). The median UIC was significantly (*p* = 0.001) lower among stunted pupils (17.0 µg/L) compared with normal children (22.0 µg/L). The proportion of stunted pupils with a UIC below the median of 17.0 µg/L (9.9%, 160) was significantly higher (*p* = 0.001) compared with those pupils with UIC above the median (5.5%, 78).

Table 2 shows the univariate and multivariable analysis of factors associated with stunting. The table shows that in a logistic regression model, females (aOR = 1.348; 95%CI: 1.006–1.905), pupils having clinical goiter (aOR = 1.739; 95% CI: 1.222–2.475), and students having UIC of less than 17µg/L (aOR = 1.934; 95% CI: 1.457–2.571) were considerably connected to stunting.

**Figure 1:**
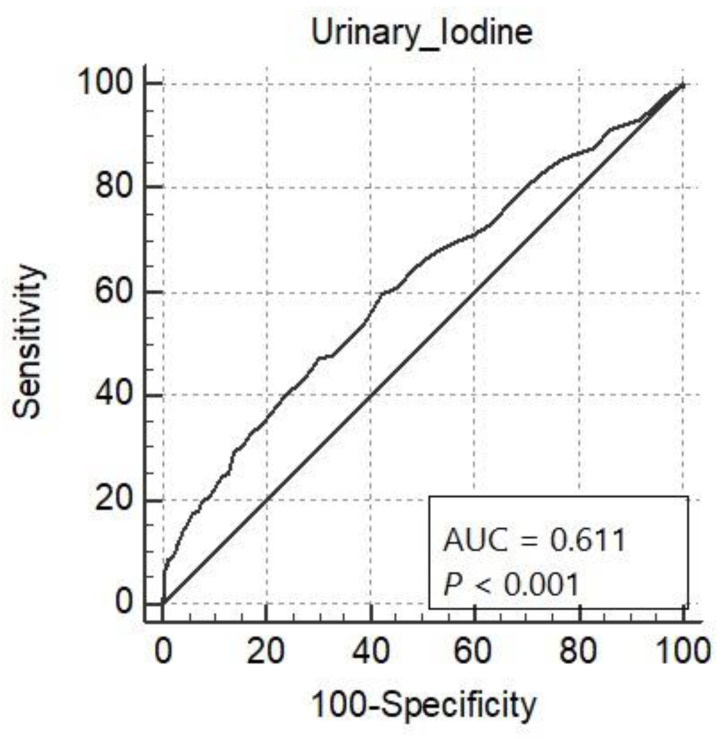
Receiver operating characteristic curve analysis for urinary iodine in µg/L to predict stunting among school children.

Table 3 and Figure 1 show ROC curve analysis of the projection of urinary iodine content for stunting. The ability of urinary iodine content to predict stunting was good (AUC = 0.611, 95% CI: 0.594–0.629). The ROC curve identified the optimum cutoff point of urinary iodine content to be ≤19.0 µg/L. The sensitivity was 59.66% (95% CI: 53.1–66.0) and the specificity was 57.62% (95% CI: 55.8–59.5).

## 4. Discussion

The present study showed a prevalence of stunting of 7.8% (95% CI: 6.9–8.8%) among school-aged children, indicating a mild public health problem (stunting prevalence of less than 20%) [21]. Similar figures of stunting were reported in Cameroon (7.5%) [22]. Lower figures of stunting were reported in Iran (1.6%) [23] and Guinea-Bissau (6.4%) [24]. On the other hand, higher figures of stunting were reported in South Africa (13.7%) [25], Haiti (14.1%) [26], Nigeria (15.4%) [27], Ethiopia (46.1%) [28], and India (64%) [29]. In Hail, Saudi Arabia, a recent study revealed a prevalence of 6.7% of stunting among school children [30]. A recent meta-analysis study including 65 low and middle-income counties using data from demographic health surveys found a pooled estimate of stunting of 29% [31].

Our results among pupils aged 8 to 10 years in the Aseer region of Saudi Arabia showed an overall prevalence of TGR of 24.0% by clinical examination. The study revealed moderate public health problems of IDD (20% to less than 30%) by WHO guidelines [32]. The Saudi countrywide study in 1995 found a goiter prevalence rate of 30% in the Aseer area. Alternatively, a recent study in 2012 described a goiter prevalence rate in Jazan (a southwestern region of Saudi Arabia) of 11% [33]. The median UIC (17.0 µg/L) of the current survey discovered a severe iodine deficiency situation [32] among school children (<20.0 µg/L).

A study in an iodine-deficient area in India (Gujarat, Western India) reported high stunting figures of 64% [29]. This study emphasized the significance of tackling the essential significance of iodine supplementation and the importance of focus on nutritional intake in general. Similarly, in primary school children in Obukpa, a rural Nigerian community, a stunning figure of 25% was found in a community of severe iodine deficiency (<20.0 µg/L) [27].

Our study disclosed that pupils having clinical goiter and students having UIC of less than 17µg/L were considerably connected with stunting. Similarly, the ability of urinary iodine content to predict stunting was good. The present study revealed that IDD was a predictor of stunting.

The role of goiter and IDD in developing stunting is most likely multifactorial. In stunted young people, iodide is less effectively captivated against the electrochemical gradient needing resources [29,34]. Iodine intensity of the thyroid gland diminishes due to low iodide clearance and uptake in stunting [35]. Stunting incidentally causes modifications in iodine metabolism that may lead to thyroid hyperplasia and decreases circulating thyroid hormone amounts. It may lead to goitrogenesis promptly via the absence of substrate accessibility, in particular the absence of essential amino acids such as tyrosine [36]. A meta-analyses study of the consequences of iodine repletion on growth displayed a convinced influence in moderate-to-mildly iodine-deficient schoolchildren on insulin-like growth factor-1 and insulin-like growth factor binding protein-3 [37].

Suitable diet and a healthful way of life are fundamental all through the life cycle to guarantee the most favorable health for both the person and future descendants. When a child ignores these, they may acquire stunting. Stunting is the second encountered form of malnutrition globally after anemia and a truly invalidating clinical condition. Stunting is the most apparent indication of a complex syndrome also involving reduced immune function, retarded growth, diminishing of cognitive function, and other metabolic disorders that might affect the individual either immediately or in the long term. The stunting syndrome is one of the leading forms of the extensive shortage of micronutrients in the diet, called “hidden hunger” [38].

Control of hidden hunger and its health-related effects are important public health concerns that can only be attained by safeguarding acceptable micronutrient consumptions in all clusters. Overall enhancement of living values is required, and dietary modification should be practiced in the long term. Immediate measures might include intensive marketing of exclusive breastfeeding up to 6 months and appropriate presentation of complementary foods [39]. Similarly, general availability of iodized salt is required for IDD elimination. Unfortunately, a recent study showed that the use of insufficient iodized salt in the Aseer region is still common [40]. Nevertheless, a systematic review paper showed that an iodine supplementation has only a marginal effect on growth, but especially in very short populations [41]. Finally, it should be noted that stunting is not a synonym of malnutrition [42]. Rather, interaction between the biology of human development and the social, economic, political, and emotional (SEPE) environment should be considered. SEPE factor regulation of human growth is shown to be a more inclusive justification for malleability in height than usual beliefs such as socioeconomic status and genetic factors [43].

Limitations of the study are mainly related to the lack of information on nutrition and differences in the diets of the study children.

## 5. Conclusions

The present study showed that stunting among school-aged children presents a mild public health problem. On the other hand, the study disclosed a severe iodine deficiency state among school children in the Aseer region. Continuous monitoring of iodine status among school children is therefore necessary. Multi-sectoral methodologies which join nutrition-sensitive with nutrition-specific approaches are expected to have a more significant influence on decreasing stunting. In the Aseer region, banning of non-iodized salt in the local markets should be strictly implemented.

## Figures and Tables

**Table 1:** Sociodemographic, clinical, and biochemical correlates of stunting among school children (*n* = 3046).

Variable	Stunting		*p*
	Absent	Present	
	(HAZ ≥ −2)	(HAZ < −2)	
Gender: no (%)			
Males	1392 (92.7%)	109 (7.3%)	0.17
Females	1416 (91.7%)	129 (8.3%)	
Goiter: no (%)			
Normal	2147 (92.7%)	169 (7.3%)	0.037 *
Enlarged (Grade I and II)	661 (90.5%)	69 (9.5%)	
Median UIC in µg/L	22	17	0.001 *
Iodine deficiency: no (%)			
Below Median UIC (<17 µg/L)	1456 (90.1%)	160 (9.9%)	0.001 *
Above Median UIC (≥17 µg/L)	1352 (94.5%)	78 (5.5%)	

*p* was calculated using Chi-square and Mann–Whitney U tests. HAZ = Height-for-age z score; * Significant (*p* < 0.05).

**Table 2:** Univariate and multivariable analysis for factors associated with stunting among school children (*n* = 3046).

Variable	cOR (95% CI)	aOR (95% CI)
Females vs. males	1.163 (0.892–1.517)	1.348 (1.006–1.905)
Clinical goiter vs. normal	1.326 (0.989–1.778)	1.739 (1.222–2.475)
UIC Below median (<17 µg/L) vs. above median (>17 µg/L)	1.904 (1.438–2.518)	1.934 (1.457–2.571)

cOR = crude odds ratio, aOR = adjusted odds ratio, 95% CI = 95% confidence interval.

**Table 3:** Results of receiver operating characteristics (ROC) for urinary iodine in µg/L to predict stunting among school children.

Condition	AUC (95% CI)	Optimal Cutoff Point of Urinary Iodine (µg/L)	Sensitivity % (95% CI)	Specificity % (95% CI)
Stunting	0.611 (0.594–0.629)	≤19	59.66 (53.1–66.0)	57.62 (55.8–59.5)

## Data Availability

Data are available on request from corresponding author.
